# Novel Echarate Virus Variant Isolated from Patient with Febrile Illness, Chanchamayo, Peru

**DOI:** 10.3201/eid2909.230374

**Published:** 2023-09

**Authors:** Gilda Troncos, Dina Popuche, Bishwo N. Adhikari, Kyle A. Long, Jane Ríos, Michel Valerio, Carolina Guevara, Regina Z. Cer, Kimberly A. Bishop-Lilly, Julia S. Ampuero, Maria Silva, Cristhopher D. Cruz

**Affiliations:** US Naval Medical Research Unit SOUTH, Lima, Peru (G. Troncos, D. Popuche, J. Ríos, C. Guevara, J.S. Ampuero, M. Silva, C.D. Cruz);; Defense Threat Reduction Agency, Fort Belvoir, Virginia, USA (B.N. Adhikari);; Biological Defense Research Directorate, Naval Medical Research Command, Fort Detrick, Maryland, USA (B.N. Adhikari, K.A. Long, R.Z. Cer, K.A. Bishop-Lilly);; Leidos, Reston, Virginia (K.A. Long);; Hospital Regional Docente de Medicina Tropical Julio César Demarini Caro, Chanchamayo, Peru (M. Valerio);; Universidad Peruana Cayetano Heredia, Lima (M. Valerio)

**Keywords:** phlebovirus, Candiru complex, Echarate, viruses, vector-borne infections, fever, next-generation sequencing, Chanchamayo, Peru

## Abstract

A new phlebovirus variant was isolated from an acute febrile patient in Chanchamayo, Peru. Genome characterization and p-distance analyses based on complete open reading frames revealed that the virus is probably a natural reassortant of the Echarate virus (large and small segments) with a yet-unidentified phlebovirus (M segment).

The genus *Phlebovirus* (order Bunyavirales, family Phenuiviridae) consists of 66 species according to the International Committee on Taxonomy of Viruses (*1*). Phleboviruses are globally distributed and can be transmitted by phlebotomine sandflies, mosquitoes, or ticks (*2*,*3*). Sandfly phlebovirus can cause unspecific symptoms in humans and often is misdiagnosed as dengue fever, malaria, or influenza (*4*,*5*); however, its clinical symptoms can range from high fever, severe headache, muscle pain, and aseptic meningitis to mild or severe meningoencephalitis (*6*). In Peru, 3 of 9 phleboviruses that cause febrile illness in Central and South America (*3*–*5*,*7*) have been identified: Echarate virus (ECHV), Maldonado virus (*7*), and Candiru virus (*7*).

During the last decade, isolates characterized by whole-genome sequencing have contributed to increased detection of novel and recombinant pathogenic and nonpathogenic phleboviruses worldwide (*2*,*5*,*7*), demonstrating a high viral diversity within this genus. Therefore, continuous public health surveillance, including genome characterization as a complementary tool, is critical to identifying novel and emerging viruses of clinical relevance in the Americas. We report the identification and characterization of a novel ECHV virus variant isolated from a patient with acute febrile illness (AFI) in Peru.

## The Study

**Figure 1:**
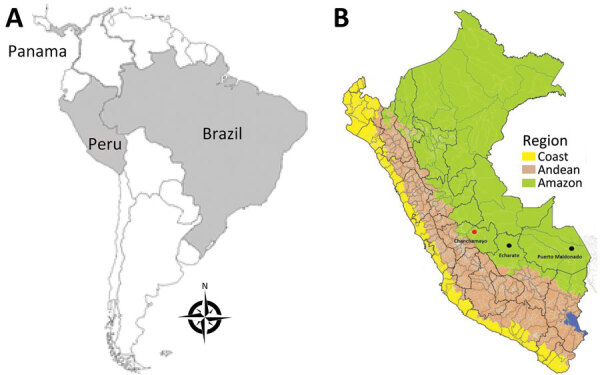
Geographic distribution of Candiru complex virus in Central and South America in study of novel ECHV variant isolated from patient with febrile illness, Chanchamayo, Peru. A) Countries where viruses were identified (shaded in gray). B) Geographic distribution of the Candiru complex viruses in Peru identified from patients with acute febrile illness. Red dot indicates location of the novel ECHV variant: ECHV variant (Chanchamayo–Junín), ECHV (Echarate-Cuzco, 1998); Maldonado virus (Puerto Maldonado–Madre de Dios, 2004); Candiru virus (Puerto Maldonado–Madre de Dios, 2010). ECHV, Echarate virus.

As part of passive clinic-based surveillance for AFI in Peru approved by Peru’s Ministry of Health and the US Naval Medical Research Unit South Institutional Review Board (protocol no. NMRCD.2010.0010) (*8*), a 20-year-old man who worked in civil construction was admitted to Hospital Regional Docente de Medicina Tropical Julio César Demarini Caro, located in the city of Chanchamayo in the northern region of Junín Department in central Peru, on June 25, 2019 (Figure 1). He had a 2-day history of fever, malaise, chills, systemic muscle pain, arthralgias, generalized head pain, drowsiness, photophobia, retroocular pain, and anorexia. He had conjunctival injection and an axillary temperature of 39.0°C, and the tourniquet test was negative.

We inoculated the acute serum sample into African green monkey kidney cells (Vero ATCC CCL-81) and *Aedes albopictus* mosquito cells (C6/36) and maintained them at 37°C (Vero cells) and 33°C (mosquito cells). The sample showed ≈50% cytopathic effect at day 7 in Vero cells, but no cytopathic effect occurred in C6/36 cells after 10 days. We prepared spot slides of both cell lines and tested them by indirect immunofluorescence assay (IFA) using pooled polyclonal antisera against flaviviruses (yellow fever virus and dengue virus serotype 3), alphaviruses (Mayaro virus and Venezuelan equine encephalitis virus), orthobunyaviruses (Oropouche virus, Guaroa virus, Caraparu virus, and Maguari virus), arenaviruses (Allpahuayo virus and Tacaribe virus), and cardiovirus. Only the Vero cells spot slide was reactive by IFA (≈25% of cells fluoresced) with pooled bunyaviruses polyclonal antibody. The second IFA with individual polyclonal antibody components also detected a weak reaction (≈25% of cells fluoresced) with Oropouche and Maguari polyclonal antibodies. Because of a weak positive signal at this level, we submitted the isolate for molecular characterization.

We extracted RNA from infected Vero cell supernatant by using the QIAamp Viral RNA Mini Kit (QIAGEN, https://www.qiagen.com), according to the manufacturer’s instructions. We amplified the viral genome by using 2 unbiased approaches, a modified sequence-independent, single-primer amplification (SISPA) protocol (*9*), and whole-transcriptome amplification (WTA) (*10*) using REPLI-g WTA Single Cell Kit (QIAGEN) according to manufacturer’s guidelines. We prepared libraries by using Nextera XT DNA Library Preparation Kit (Illumina, https://www.illumina.com) and sequenced them on the Illumina MiSeq platform by using MiSeq Reagent Kit version 3 (600-cycle) according to the manufacturer’s instruction.

We processed raw reads from both sequencing approaches (SISPA and WTA) for quality control, de novo assembly, host read subtraction, taxonomic classification, and gene family analysis by using 3 different bioinformatics pipelines: EDGE Bioinformatics tools (*11*), VirusSeeker (*12*), and MetaDetector (K.A. Bishop-Lilly et al., unpub. data). Both unbiased methods showed similar read quality. Results of taxonomic analysis of the reads and contigs obtained from SISPA and WTA showed Candiru phlebovirus as the unique human viral pathogen in the isolate, indicating that both techniques successfully amplified the isolated virus. We searched consensus sequences (GenBank accession nos. OQ623470–2) against a nucleotide database by using BLASTn and protein database by using BLASTx (both at https://blast.ncbi.nlm.nih.gov). Candiru phlebovirus large and small segments had >95% amino acid identity compared with those of ECHV. Of note, the medium (M) segment had 76.5% identity with that of ECHV at nucleotide level and 86.36% identity at amino acid level (Table 1). The M segment typically encodes for 3 polypeptides (NSm, Gn, and Gc), which are co-translationally cleaved. The NSm polypeptide is a virulence factor associated with the inhibition of apoptosis in infected cells and plays a role in viral mosquito infection (*13*). The amino acid identity value of the predicted NSm sequence of our isolated virus ranged from <30% with the other members of Candiru complex to 78.6% with ECHV. For average coverage calculation, we mapped the trimmed reads back to contigs obtained from de novo assembly and to the corresponding Echarate reference sequences as an orthogonal verification at 0.8 length fraction and 0.8 similarity fraction. The minimum coverage was 1,583× in the M segment with ECHV and the maximum was 10,224× in the M segment with the obtained contig (Table 2).

**Table 1:** Summary of nucleotide and amino acid similarity for a novel Echarate virus variant isolated from patient with febrile illness, Chanchamayo, Peru*

Segment	% Nucleotide identity; % coverage (accession no.)	% Amino acid identity (accession no.)
Large	83.2; 99 (HM119410.1)	97.01 (AEA30058.1)
Medium	76.5; 97† (HM119411.1)	86.36 (AEA30046.1)
Small	91.32; 100 (HM119412.1)	96.37‡ (AEA30072.1); 100§ (AEA30073.1)

*Accession nos. represent best hits on GenBank. †Result after a BLASTn (https://blast.ncbi.nlm.nih.gov) search was optimized for more dissimilar sequences (discontiguous megablast). ‡Nonstructural. §Nucleoprotein.

**Table 2:** Depth and breadth of coverage based on reads mapped to references for a novel Echarate virus variant isolated from patient with febrile illness, Chanchamayo, Peru*

Segment	Reference	No. mapped reads	Depth coverage	Breadth coverage, %
Large	Contig	111,471	8,163.64×	100
	ECHV (HM119410, 6,411 bp)	201,837	4,777.26×	100
Medium	Contig	164,685	10,224.69×	100
	ECHV (HM119411, 4,287 bp)	24,756	1,583.69×	48
Small	Contig	20,585	6,358×	100
	ECHV (HM119412,1,818 bp)	24,690	3,561.94×	100

*GenBank accession numbers and sequence length for each ECHV segment are in parentheses. ECHV, Echarate virus.

We performed pairwise sequence comparison between our isolate and Candiru complex viruses for the RNA-dependent RNA polymerase, glycoprotein precursor, nucleoprotein, and nonstructural genes. The p-distance value of the glycoprotein precursor gene 0.237 (nucleotide) between our isolate and ESCV was similar to those observed among other members of the Candiru complex (Appendix 1 Table) and consistent with values previously reported in the literature among members of the Candiru complex (0.2–0.46) and among members of the same complex but not among strains of the same virus (0.01–0.12) (*7*,*14*,*15*). Furthermore, considering the new variant was isolated and characterized 21 years later, we also calculated the overall mean distance values (0.079 for nucleotide, 0.039 for amino acid) for 74 complete M segment sequences of Oropouche virus published over time (1955–2021 [67 years]). Those values suggest that the difference could not be explained by virus mutation because the new isolate has a nucleotide difference value of 0.24 with ECHV. The low M segment identity value together with distance values probably indicate the uniqueness of this segment and support the concept that this is a novel ECHV variant that could be generated by a recombinant event between ECHV and an unknown phlebovirus. 

**Figure 2:**
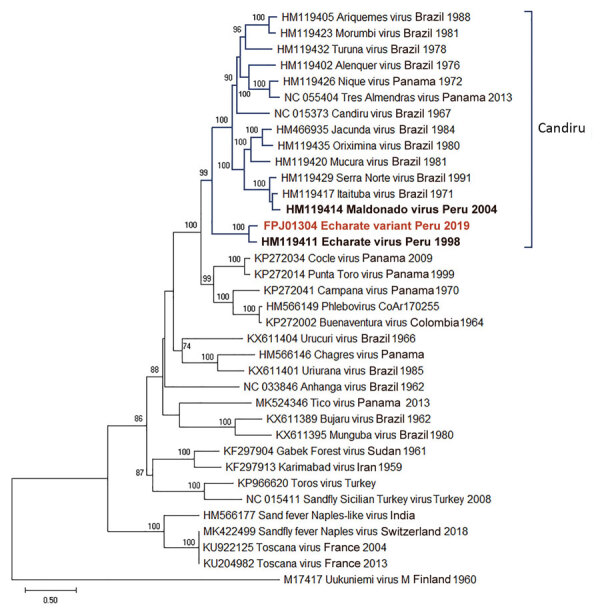
Maximum-likelihood phylogenetic tree based on 36 amino acid sequences of phleboviruses M segment (NSm–Gn) in study of novel Echarate virus variant isolated from patient with febrile illness, Chanchamayo, Peru. Strains from Peru are in bold, and the novel variant is in red. Only bootstrap values >70% are shown at key nodes. Uukuniemi virus was considered as the outgroup. Scale bar indicates nucleotide substitutions per site.

To determine the evolutionary relationship of our isolate to other known members of the genus, we conducted maximum-likelihood phylogenetic analyses on the aligned amino acid sequence of the RNA-dependent RNA polymerase, glycoprotein precursor, nucleoprotein, and nonstructural genes (Appendix 2). All the phylogenetic trees placed our isolate among the Candiru virus complex within a well-supported clade with ECHV. However, the NSm or glycoprotein tree clustered the new variant together with ECHV within a well-supported clade separate from other Candiru complex viruses (Figure 2).

## Conclusions

Our findings indicate that a novel ECHV variant is circulating in the jungle of central Peru. Because the clinical symptoms of infection with this variant are also characteristic of dengue, malaria, and other tropical infectious diseases common in this region (*4*,*5*) continued AFI biosurveillance is needed to detect novel and emerging pathogens to protect the health of the population and US service members deployed in affected areas in Peru. Ecologic studies are necessary to determine how widespread the new variant is within this region, to identify potential vectors and reservoirs involved in its transmission, and to support decision-making for keeping service members medically prepared and protected from health and safety threats both on and off duty.

Appendix 1 Pairwise distance matrix of genomes in a study of novel Echarate virus variant isolated from patient with febrile illness, Chanchamayo, Peru.

Appendix 2 Additional information about novel Echarate virus variant isolated from patient with febrile illness, Chanchamayo, Peru.
