# Supplementary material for: Novel Echarate Virus Variant Isolated from Patient with Febrile Illness, Chanchamayo, Peru
Source: Emerg Infect Dis. 2023 Sep;29(9):1908–12. doi: 10.3201/eid2909.230374 (PMC10461681; doi:10.3201/eid2909.230374)
Supplement: Appendix 2 — Additional information about novel Echarate virus variant isolated from patient with febrile illness, Chanchamayo, Peru. [file 23-0374-Techapp-s2.pdf]

Article DOI: https://doi.org/10.3201/eid2909.230374

EID cannot ensure accessibility for supplementary materials supplied by authors. Readers who have difficulty accessing supplementary content should contact the authors for assistance.

Novel Echarate Virus Variant Isolated from Patient with Febrile Illness, Chanchamayo, Peru

Appendix 2

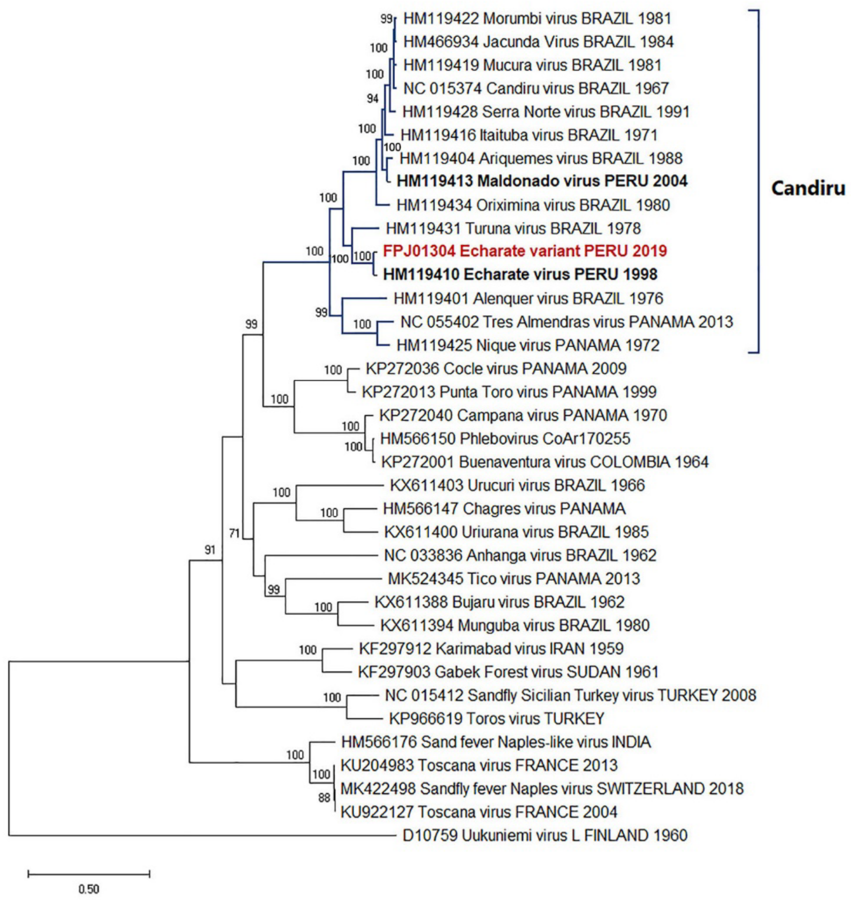

Appendix 2 Figure 1. Maximum-likelihood phylogeny based on 36 aa sequences of phleboviruses L segment (RdRp). Peruvian strains are in bold and the novel variant is highlighted in red. Only bootstrap values >70% are shown at key nodes. Uukuniemi virus was considered as the outgroup. Scale bar indicates nucleotide substitutions per site. aa, amino acid; L, large segment; RdRp, RNA-dependent RNA polymerase

A

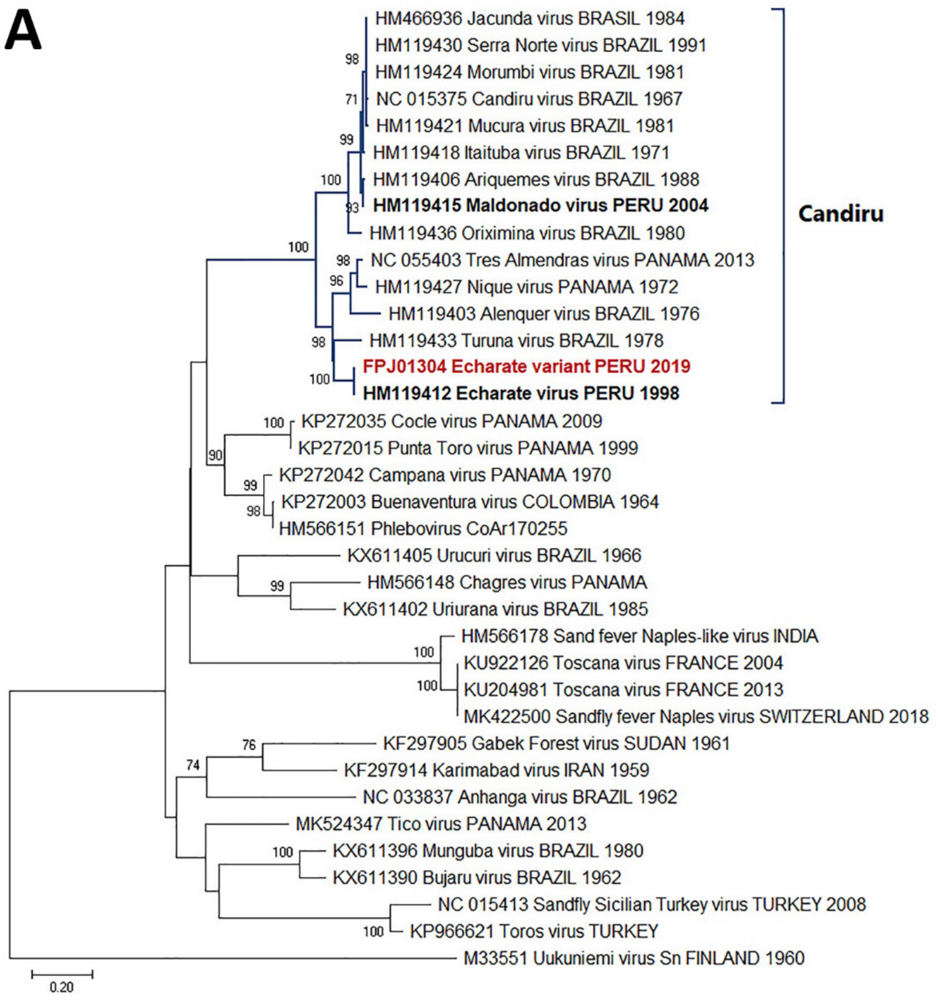
HM466936 Jacunda virus BRASIL 1984
HM119430 Serra Norte virus BRAZIL 1991
HM119424 Morumbi virus BRAZIL 1981
NC 015375 Candiru virus BRAZIL 1967
HM119421 Mucura virus BRAZIL 1981
HM119418 Itaituba virus BRAZIL 1971
HM119406 Ariquemes virus BRAZIL 1988
HM119415 Maldonado virus PERU 2004
HM119436 Oriximina virus BRAZIL 1980
NC 055403 Tres Almendras virus PANAMA 2013
HM119427 Nique virus PANAMA 1972
HM119403 Alenquer virus BRAZIL 1976
HM119433 Turuna virus BRAZIL 1978
FPJ01304 Echarate variant PERU 2019
HM119412 Echarate virus PERU 1998
Candiru
KP272035 Cocle virus PANAMA 2009
KP272015 Punta Toro virus PANAMA 1999
KP272042 Campana virus PANAMA 1970
KP272003 Buenaventura virus COLOMBIA 1964
HM566151 Phlebovirus CoAr170255
KX611405 Urucuri virus BRAZIL 1966
HM566148 Chagres virus PANAMA
KX611402 Uriurana virus BRAZIL 1985
HM566178 Sand fever Naples-like virus INDIA
KU922126 Toscana virus FRANCE 2004
KU204981 Toscana virus FRANCE 2013
MK422500 Sandfly fever Naples virus SWITZERLAND 2018
KF297905 Gabek Forest virus SUDAN 1961
KF297914 Karimabad virus IRAN 1959
NC 033837 Anhanga virus BRAZIL 1962
MK524347 Tico virus PANAMA 2013
KX611396 Munguba virus BRAZIL 1980
KX611390 Bujaru virus BRAZIL 1962
NC 015413 Sandfly Sicilian Turkey virus TURKEY 2008
KP966621 Toros virus TURKEY
M33551 Uukuniemi virus Sn FINLAND 1960
98
71
99
100
93
100
98
96
98
100
100
90
99
98
99
100
100
76
74
100
100
0.20

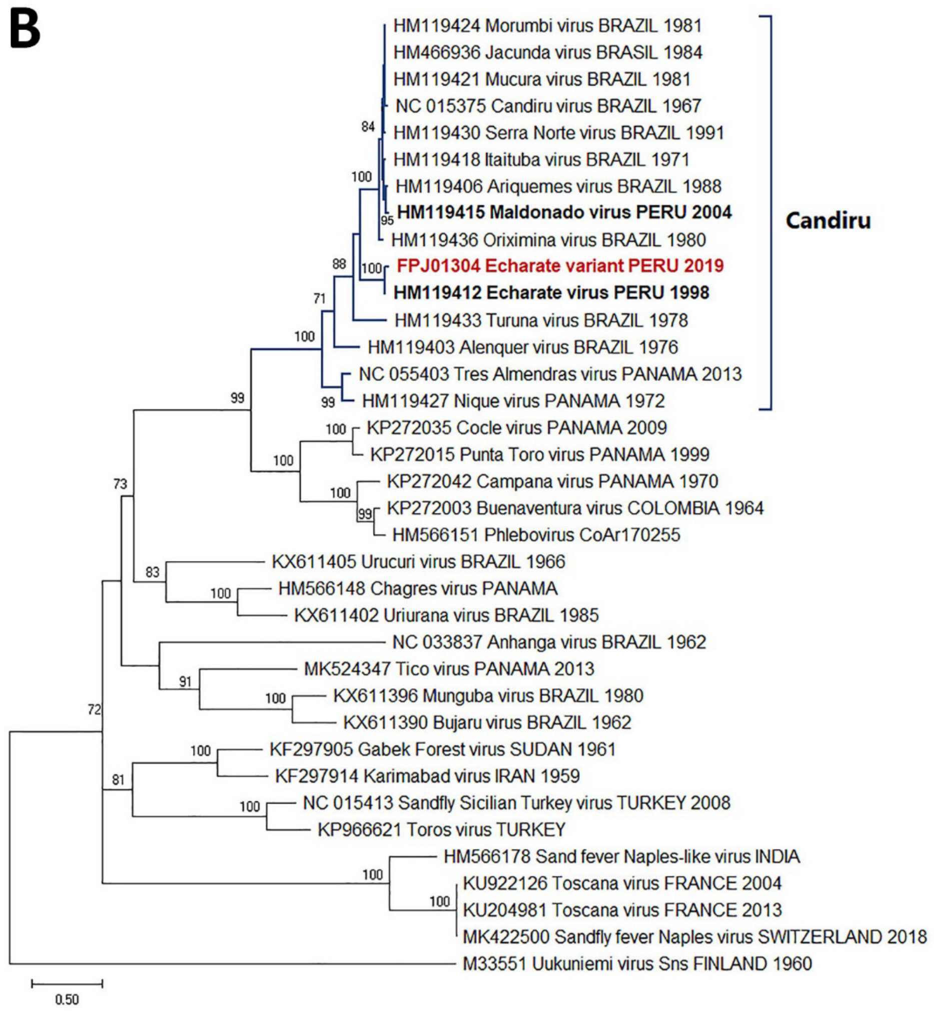

Appendix 2 Figure 2. Maximum-likelihood phylogeny based on 36 aa sequences of phleboviruses S segment. A) Nucleocapsid protein; B) Nonstructural protein. Peruvian strains are in bold and the novel variant is highlighted in red. Only bootstrap values >70% are shown at key nodes. Uukuniemi virus was considered as the outgroup. Scale bar indicates nucleotide substitutions per site. aa, amino acid; S, small segment.
